# Abdominal Clonus as a Rare Visceromotor Manifestation in Spinal Cord Injury: A Case Report

**DOI:** 10.7759/cureus.116313

**Published:** 2026-09-15

**Authors:** Sofia Mesquita, Arminda Lopes, Catarina S Silva, Joao P Paiva, Hipólito Nzwalo

**Affiliations:** 1 Physical Medicine and Rehabilitation, Centro de Medicina de Reabilitação do Sul, Faro, PRT; 2 Stroke Unit, Algarve University Hospital Center, Faro, PRT; 3 Faculty of Medicine and Biomedical Sciences, University of Algarve, Faro, PRT

**Keywords:** abdominal clonus, autonomic dysreflexia, bladder distension, neurogenic bladder, spinal cord injury, visceromotor reflex

## Abstract

Spinal cord injury can lead to reorganization of spinal reflex circuits and loss of supraspinal modulation of autonomic and somatic functions. Although bladder distension is a well-recognized trigger of autonomic dysreflexia, associated somatic motor manifestations are rarely described.

We report the case of a 69-year-old man with a long-standing traumatic complete C6 spinal cord injury and chronic neurogenic bladder. Neurological classification according to the International Standards for Neurological Classification of Spinal Cord Injury (ISNCSCI) was American Spinal Injury Association (ASIA) Impairment Scale (AIS) grade A. He experienced daily episodes of involuntary rhythmic abdominal contractions that reproducibly occurred in temporal association with bladder filling and ceased promptly after bladder emptying. In the absence of conscious sensation below the level of injury, the patient recognized these contractions as a sign of bladder fullness. When abdominal clonus occurred, he reclined his wheelchair to a near-supine position, facilitating reflex voiding into an external urinary collection device, with immediate cessation of the contractions following bladder emptying. His medical history included previous episodes of autonomic dysreflexia associated with bladder distension and marked structural changes related to chronic neurogenic bladder.

This phenomenon may reflect aberrant recruitment of somatic motor circuits by visceral bladder afferents in the setting of chronic spinal hyperexcitability. In this case, abdominal clonus acquired an adaptive functional role, providing a clinically useful warning sign of bladder fullness in a patient at risk of autonomic dysreflexia. Abdominal clonus may represent a rare visceromotor manifestation of bladder distension after complete spinal cord injury. Recognition of such reflex motor responses may have clinical relevance for neurogenic bladder monitoring and the prevention of autonomic complications.

## Introduction

Micturition depends on coordinated integration of peripheral sensory input, spinal reflex circuits, and supraspinal centers in the brainstem and cerebral cortex [1-4]. Lower urinary tract storage and emptying are mediated by sympathetic pathways arising from T11-L2 via the hypogastric nerve, parasympathetic pathways arising from S2-S4 via the pelvic nerve, and somatic control of the external urethral sphincter through the pudendal nerve (S2-S4) [1,2]. During bladder filling, afferent signals generated by increasing bladder-wall tension ascend to supraspinal centers, allowing conscious perception of fullness and coordinated initiation of voiding [1-4].

Suprasacral spinal cord injury disrupts this integration and can produce neurogenic lower urinary tract dysfunction, including detrusor overactivity and detrusor-sphincter dyssynergia [5-7]. Over time, chronic dysfunction may be accompanied by structural bladder remodeling, including wall thickening, trabeculation and diverticular change, together with urinary stasis and recurrent urinary tract infection [1,6,7].

Cervical and high thoracic spinal cord injury may also lead to autonomic dysreflexia, in which afferent stimuli below the level of injury provoke an exaggerated sympathetic response; bladder distension is the most frequent urological trigger [8-10]. Clonus is characterized by involuntary rhythmic muscle contractions related to hyperexcitability of spinal stretch-reflex pathways after an upper motor neuron lesion and is distinct from sustained velocity-dependent hypertonia or non-rhythmic spasms [11]. It most commonly affects distal muscle groups, whereas abdominal clonus is rarely reported, including after spinal cord injury [12].

Chronic spinal cord injury is associated with plastic changes in spinal reflex pathways, and bladder afferent activity can modulate somatic spinal reflexes [13-15]. We report a patient with a long-standing complete C6 spinal cord injury who developed recurrent bilateral rhythmic abdominal wall contractions in temporal association with bladder filling, with cessation after bladder emptying. The case illustrates an unusual motor phenomenon that the patient used as a surrogate warning sign of bladder fullness in the absence of conscious visceral sensation.

## Case presentation

We present the case of a 69-year-old man with tetraplegia secondary to a traumatic spinal cord injury sustained in 1974. The patient has a C6 neurological level of injury, classified as International Standards for Neurological Classification of Spinal Cord Injury (ISNCSCI) American Spinal Injury Association (ASIA) Impairment Scale (AIS) grade A.

His medical history is notable for chronic neurogenic bladder complicated by recurrent urinary tract infections, and he had previously undergone right nephrectomy, ureterotomy, and sphincterotomy in 2006. His medical history also includes type 2 diabetes mellitus, obstructive sleep apnea syndrome treated with continuous positive airway pressure (CPAP), and osteoporosis. His urological treatment regimen includes prophylactic nitrofurantoin and an Escherichia coli bacterial lysate.

Serial imaging assessments revealed structural changes consistent with long-standing neurogenic bladder, including marked bladder-wall thickening and irregularity, trabeculation, heterogeneous intravesical contents associated with chronic urinary stasis, and persistent post-void residual urine (Figures 1, 2).

**Figure 1:**
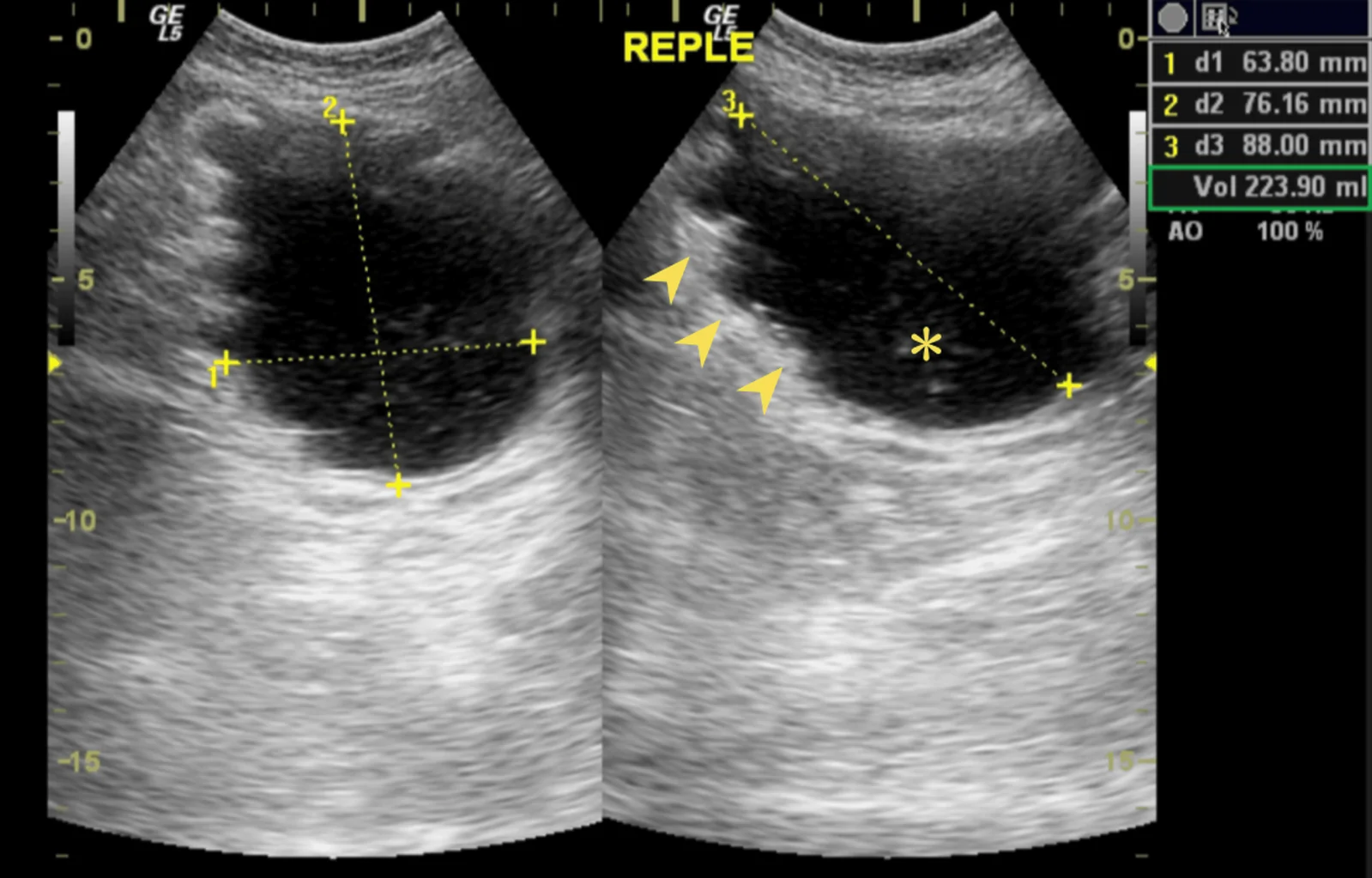
Ultrasound of the distended neurogenic bladder Transabdominal ultrasound obtained with the bladder distended, demonstrating marked irregularity and trabeculation of the bladder wall (arrowheads) and heterogeneous dependent echogenic intravesical material compatible with urinary sediment/debris (asterisk). The estimated bladder volume was 223.9 mL.

**Figure 2:**
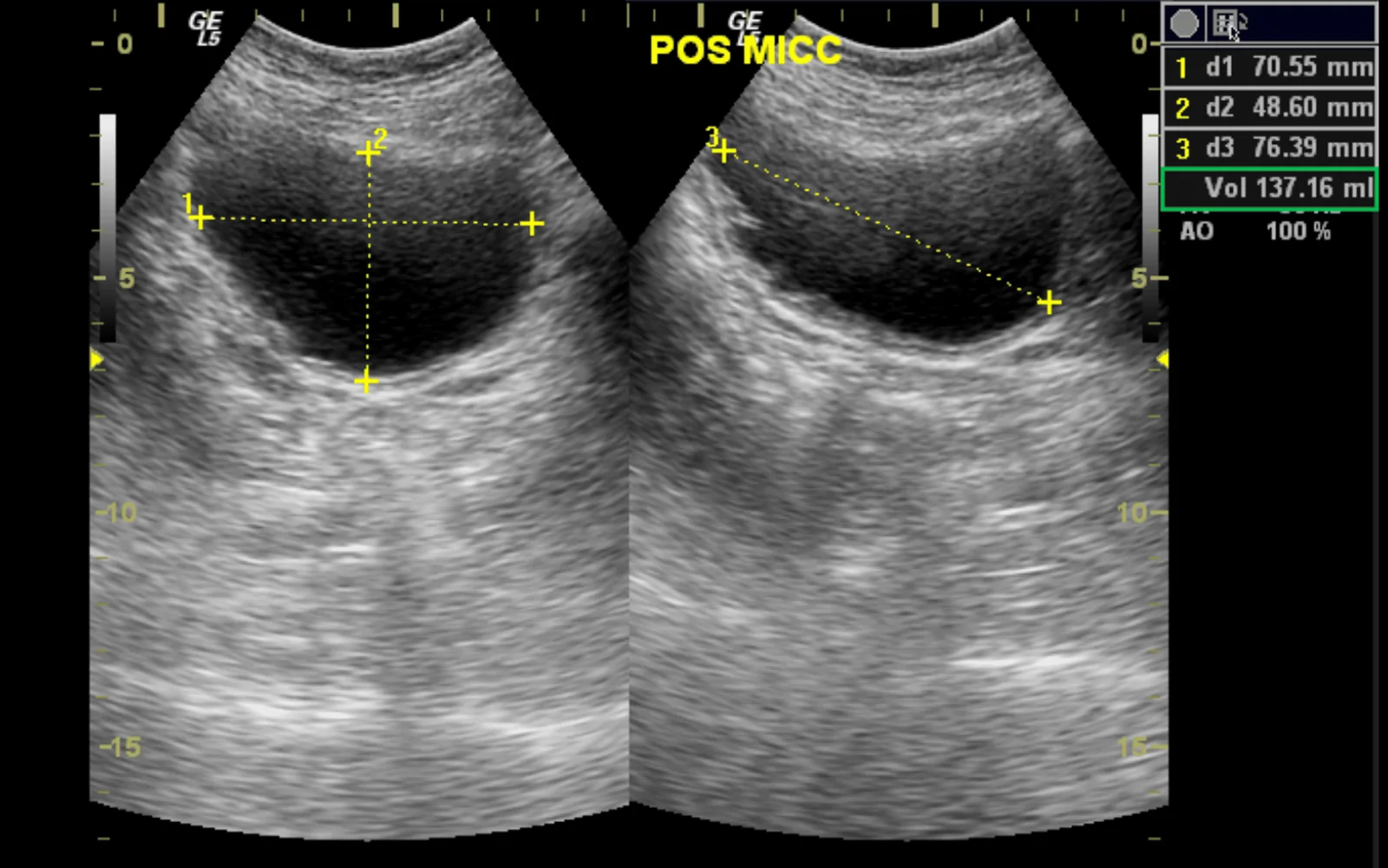
Post-void residual urine in chronic neurogenic bladder Post-void transabdominal ultrasound demonstrating persistent residual urine, with an estimated bladder volume of 137.2 mL. Irregularity of the bladder wall and heterogeneous dependent intravesical echoes compatible with urinary sediment remain visible.

The ultrasound-derived bladder volumes reported in Figure 1 and Figure 2 were obtained during routine imaging and were not synchronized with an abdominal clonus episode; therefore, they should not be interpreted as defining a volume threshold for the onset or resolution of the contractions.

No recent urodynamic assessment was available as part of the present evaluation even though such testing could have provided additional information regarding bladder compliance, detrusor pressures, and detrusor-sphincter coordination.

The patient experiences daily episodes of involuntary rhythmic contractions of the abdominal wall. The contractions were bilateral and approximately symmetric, predominantly involving the anterior abdominal wall (Video 1).

**Video 1:** Abdominal clonus Rhythmic involuntary abdominal wall contractions consistent with abdominal clonus in a patient with complete C6 spinal cord injury

Because electromyography was not performed, the specific abdominal muscles generating the contractions could not be determined. A reproducible temporal pattern was observed, with the contractions occurring after a period of bladder filling, preceding reflex voiding, and ceasing promptly after bladder emptying. According to the patient and repeated clinical observation, this sequence recurred consistently over time; however, the number of individual episodes was not prospectively recorded. The bladder volume and intravesical pressure at the onset of the contractions were not objectively quantified, as no simultaneous urodynamic assessment was performed during an episode. The patient recognizes this motor phenomenon as the functional equivalent of bladder fullness in the absence of conscious visceral sensation below the level of injury. When the clonus develops, the patient uses a postural strategy that he has developed empirically over the years: he electrically reclines his wheelchair to a near-supine position, thereby facilitating reflex voiding into an external urinary collection device. Following bladder emptying, the abdominal contractions cease.

During previous hospital admissions, episodes of autonomic dysreflexia associated with bladder distension were documented. The patient currently manages bladder emptying through reflex voiding with an external urinary collection device, using a specific position in his powered wheelchair, because of his social circumstances and personal preference.

## Discussion

The central observation in this case is the repeatedly observed temporal association between bladder filling, onset of rhythmic abdominal contractions, and cessation of the contractions after bladder emptying. Loss of supraspinal inhibitory control and chronic spinal plasticity after spinal cord injury can amplify autonomic and motor reflex responses [5,6,13,14]. Bladder afferent input can also modulate somatic spinal reflexes [15], providing a plausible basis for aberrant recruitment of abdominal motor pathways. However, this proposed visceromotor mechanism was not directly demonstrated in the present case.

Long-standing neurogenic lower urinary tract dysfunction may have provided an important physiological context for the phenomenon. Serial imaging demonstrated marked bladder-wall thickening and trabeculation, chronic urinary stasis, and persistent post-void residual urine. Although detrusor overactivity, detrusor-sphincter dyssynergia, reduced bladder compliance, and elevated intravesical pressures are well-recognized after suprasacral spinal cord injury [6,7], these functional abnormalities and their severity were not objectively characterized in this patient by recent urodynamic assessment.

Other contributors to spinal hyperexcitability cannot be excluded. Recurrent urinary tract infection may alter bladder afferent signaling in chronic spinal cord injury [6,14], while late structural complications such as post-traumatic syringomyelia or spinal cord tethering may contribute to neurological deterioration or altered motor excitability decades after injury [16,17]. The patient also had a history of intermittent asymptomatic bacteriuria and urinary colonization; in spinal cord injury, symptomatic urinary tract infection may present atypically, including with new or worsening spasticity or autonomic dysreflexia [18]. Nevertheless, the repeatedly observed temporal sequence of bladder filling, abdominal clonus, and cessation after emptying supports bladder distension as the most plausible immediate trigger, although no specific bladder-volume threshold was established.

For this patient, the abdominal contractions acquired a practical warning function. In the absence of conscious visceral sensation below the level of injury, he recognized the contractions as a sign of bladder fullness and reclined his wheelchair to facilitate reflex voiding. He also had a separate documented history of autonomic dysreflexia associated with bladder distension, a well-recognized trigger in injuries above T6 [8-10]. Blood pressure, heart rate, and other autonomic manifestations were not systematically recorded during the abdominal clonus episodes described here; therefore, it cannot be determined whether clonus occurred independently of, concurrently with, or before autonomic dysreflexia. Bladder emptying nevertheless removes a recognized trigger of autonomic dysreflexia while also terminating the abdominal motor response in this patient.

A key limitation of this report is the absence of synchronized urodynamic and electrophysiological assessment during an abdominal clonus episode. Bladder volume and intravesical pressure at the onset of the contractions were not objectively measured, the individual abdominal muscles could not be electrophysiologically identified, and the number of episodes was not prospectively quantified. The ultrasound-derived bladder volumes were obtained during routine imaging rather than during a clonus episode and therefore do not define a triggering threshold. In addition, no recent spinal magnetic resonance imaging specifically excluded post-traumatic syringomyelia or tethering. The relationship between bladder filling and abdominal clonus should therefore be interpreted as a reproducible temporal clinical association rather than proof of causality. Future assessment using simultaneous cystometry, surface electromyography, and autonomic monitoring could provide more objective characterization.

Despite these limitations, this case suggests that unusual rhythmic motor responses may carry clinically meaningful information about visceral distension in individuals with complete spinal cord injury. Recognition of similar phenomena may help clinicians identify patient-specific warning signs and guide further objective investigation.

## Conclusions

This case suggests that rhythmic abdominal wall contractions may represent an unusual visceromotor manifestation temporally associated with bladder filling in chronic complete spinal cord injury. In this patient, the contractions repeatedly occurred during bladder filling and ceased after bladder emptying, functioning as an individual surrogate warning sign of bladder fullness in the absence of conscious visceral sensation.

Although altered spinal reflex excitability and recruitment of somatic motor pathways by visceral afferent input provide a plausible neurophysiological explanation, this mechanism was not objectively demonstrated. Recognition of similar motor phenomena may be clinically relevant in patients with spinal cord injury, particularly in those at risk of autonomic dysreflexia.
